# Gene Ontology and KEGG Pathway Enrichment Analysis of a Drug Target-Based Classification System

**DOI:** 10.1371/journal.pone.0126492

**Published:** 2015-05-07

**Authors:** Lei Chen, Chen Chu, Jing Lu, Xiangyin Kong, Tao Huang, Yu-Dong Cai

**Affiliations:** 1 College of Life Science, Shanghai University, Shanghai, People’s Republic of China; 2 College of Information Engineering, Shanghai Maritime University, Shanghai, People’s Republic of China; 3 Institute of Biochemistry and Cell Biology, Shanghai Institutes for Biological Sciences, Chinese Academy of Sciences, Shanghai, People’s Republic of China; 4 Department of Medicinal Chemistry, School of Pharmacy, Yantai University, Shandong, Yantai, People’s Republic of China; 5 Institute of Health Sciences, Shanghai Institutes for Biological Sciences, Chinese Academy of Sciences, Shanghai, People’s Republic of China; The University of Hong Kong, HONG KONG

## Abstract

Drug-target interaction (DTI) is a key aspect in pharmaceutical research. With the ever-increasing new drug data resources, computational approaches have emerged as powerful and labor-saving tools in predicting new DTIs. However, so far, most of these predictions have been based on structural similarities rather than biological relevance. In this study, we proposed for the first time a “GO and KEGG enrichment score” method to represent a certain category of drug molecules by further classification and interpretation of the DTI database. A benchmark dataset consisting of 2,015 drugs that are assigned to nine categories ((1) G protein-coupled receptors, (2) cytokine receptors, (3) nuclear receptors, (4) ion channels, (5) transporters, (6) enzymes, (7) protein kinases, (8) cellular antigens and (9) pathogens) was constructed by collecting data from KEGG. We analyzed each category and each drug for its contribution in GO terms and KEGG pathways using the popular feature selection “minimum redundancy maximum relevance (mRMR)” method, and key GO terms and KEGG pathways were extracted. Our analysis revealed the top enriched GO terms and KEGG pathways of each drug category, which were highly enriched in the literature and clinical trials. Our results provide for the first time the biological relevance among drugs, targets and biological functions, which serves as a new basis for future DTI predictions.

## Introduction

Drug-target interaction (DTI) studies are of great importance for drug research and development (R&D), as they give rise to a better understanding of how drug molecules interact with their targets and predict possible adverse drug reactions (ADRs). Over the past decade, statistics have revealed a significant decrease in the rate that new drug candidates are translated into effective therapies in the clinic [1], and drug repositioning has grown in importance. The application of known drugs and compounds for new indications would require even more DTI information. Because the experimental examination of DTI is both time- and labor-consuming, it is necessary to develop computational approaches in this field.

The use of *in silico* methods as a complement can help researchers to quickly obtain useful information. In recent years, a great deal of effort has been expended on the prediction of DTIs, and a number of methods have been developed.

Text-mining approaches emerged as a simple and convenient tool to search published literature for the associations between drugs and genes [2], but they tend to produce redundancy due to multiple gene and chemical names. Later, molecular docking approaches were widely applied in DTI studies. Cheng *et al*. used molecular docking to identify drugs and their targets [3], and Li *et al*. developed reverse ligand-protein docking to automatically search for compound-protein interactions [4]. Despite these advantages, docking and reverse docking are only suitable for proteins with known 3D structures, which limits their applications. Other computational methods predict DTIs by similarities in phenotypic side effects [5] or chemical structures [6] or by connections between chemicals with chemicals/proteins [6]. Moreover, several network-based algorithms have been applied for DTI prediction. Prado-Prado *et al*. developed multi-target QSAR (Quantitative Structure–Activity Relationship) models with 3D structural parameters and artificial neural network algorithms for the prediction of acetylcholinesterase and its inhibitors [7]. Cheng *et al*. employed network-based inference methods to identify new targets for known drugs [8].

Despite the advancement in computational methods in DTI prediction, the above methods are primarily based on the structural similarities of drugs rather than biological relevance. Recently, several studies have reported the feasible prediction of drug targets and drug repositioning using drug-involved pathway analysis. For example, Kotelnikova *et al*. found one signaling pathway that was associated with glioblastoma by retrieving references and databases and searching for compounds that affected multiple proteins in this pathway [9]. Cramer *et al*. found using molecular pathway analysis that bexarotene, an anticancer drug, may be used to treat Alzheimer’s disease [10]. Li *et al*. developed a prediction model for drug repositioning using targets and pathways based on causal chains connecting drugs to diseases [11]. In view of this, investigation of the association between pathways and drugs is helpful for discovering targets of drug compounds, thereby obtaining new drug effects. These studies made progress in the investigation of drugs with biological functions.

DrugBank (http://www.drugbank.ca/, version 4.1, accessed July 19, 2014) [12,13] contains 7,685 drug entries and 4,282 non-redundant proteins that are linked to these drug entries. The large quantity of DTI pairs is worthy of further investigation. KEGG (Kyoto Encyclopedia of Genes and Genomes) provides a drug target-based classification system in which drugs are classified into several classes according to their target proteins in KEGG DRUG (http://www.genome.jp/kegg/drug/) [14].

Here, we adapted this classification database and divided all 2,015 drugs into following nine classes based on their targets: (1) 657 drugs that target G Protein-coupled receptors (GPCRs) (*e*.*g*., Levodopa, Metoprolol and Phentolamine); (2) 35 drugs that target Cytokine receptors (CRs) (*e*.*g*., Insulin and Afatinib); (3) 228 drugs that target Nuclear receptors (NRs) (*e*.*g*., Testosterone, Estradiol and Tamoxifen); (4) 257 drugs that target Ion channels (ICs) (*e*.*g*., Nifedipine, Phenobarbital and Sertraline); (5) 37 drugs that target Transporters (Ts) (*e*.*g*., Hydrochlorothiazide and Indapamide); (6) 28 drugs that target Protein kinases (PKs) (*e*.*g*., Aspirin and Methotrexate; PKs are always downstream of GPCR, CR, IC or T in certain signaling pathways); (7) 451 drugs that target Enzymes (Es) (*e*.*g*., Metformin and Phenformin; Es represents large biological molecules that are involved in thousands of metabolic processes that sustain life); (8) nine drugs that target Cellular antigens (CAs) (*e*.*g*., imiquimod); and (9) 313 drugs that target Pathogens (Ps) (*e*.*g*., Penicillin and Levofloxacin).

If the target-based class of a given drug can be identified, its potential target proteins can be restrained to this class, thereby reducing the search area. In our previous study, a computational method was proposed to identify the target-based classes of drugs [6]. However, that study was a methodology paper that could not identify factors that contribute to the determination of drug target-based classes. In this study, we interpreted this system based on biological significance. It has been demonstrated that pathways may be important factors; additionally, Gene Ontology (GO) can represent gene product properties [15,16]. The enrichment theory was used to extract features from each pathway and each GO term to represent each investigated drug. To analyze these features, a popular feature selection method, the minimum redundancy maximum relevance (mRMR) [17], was used to evaluate each feature, thereby uncovering the important pathways and GO terms in this system. Finally, 19 key KEGG pathways and 45 key GO terms were selected to analyze the correlations between drugs and their target-based classes.

In this study, a total of 19 functionally enriched KEGG pathways and 45 functionally enriched GO terms for drug molecules were investigated for their enrichment in these target-based classes. In the remainder of this section, we provide a detailed discussion of key KEGG pathways and GO terms according to their level values in the nine target-based classes. We demonstrate that this classification scheme provides useful information for the determination of drug target-based classes.

## Materials and Methods

### Materials

The codes of 3,610 drug compounds were retrieved from our previous study [6]; this dataset originated from KEGG DRUG, one of the main databases in KEGG (http://www.genome.jp/kegg/drug/, accessed September 2012). The drugs were classified into ten classes according to the information in KEGG DRUG: (1) G protein-coupled receptors (GPCR); (2) Cytokine receptors (CR); (3) Nuclear receptors (NR); (4) Ion channels (IC); (5) Transporters (T); (6) Enzymes (E); (7) Protein kinases (PK); (8) Cellular antigens (CA); (9) Cytokines (C); and (10) Pathogens (P). Because drug compounds belonging to more than one class may produce noise and make it difficult to obtain key features, these drugs were excluded; after exclusions, a total of 3,537 classified drug compounds were obtained.

To obtain a high-quality and well-defined dataset, these 3,537 drugs were refined as follows: (I) Map 3,537 drugs with their PubChem IDs; 2,425 drug compounds had available PubChem IDs; (II) Exclude those that have no association with any human protein (this definition can be found in Section 2.2), resulting in 2,016 drugs; and (III) Exclude the class ‘Cytokines’ and the only drug (‘CID010173277’). Finally, we obtained a dataset *S* consisting of 2,015 drug compounds that were classified into nine target-based classes: (1) GPCR, (2) CR, (3) NR, (4) IC, (5) T, (6) E, (7) PK, (8) CA, and (9) P. The distribution of these 2,015 drug compounds is shown in **Table 1**. Additionally, the codes of these 2,015 drug compounds and their target-based classes are available in **S1 Table**.

**Table 1:** The distribution of the drug compounds in dataset *S*.

Class code	Target-based class	Target-based class abbreviation	Number of drug compounds
1	G protein-coupled receptors	GPCR	657
2	Cytokine receptors	CR	35
3	Nuclear receptors	NR	228
4	Ion channels	IC	257
5	Transporters	T	37
6	Enzymes	E	451
7	Protein kinases	PK	28
8	Cellular antigens	CA	9
9	Pathogens	P	313
Total	—-	—-	2,015

### Associations between chemicals and proteins

To investigate which GO terms or pathways can determine drug target-based classes, a bridge was required to associate drugs and GO terms or KEGG pathways. Human proteins are suitable because they link drug compounds and both GO terms or KEGG pathways. The linkage of proteins and GO terms or KEGG pathways can be easily obtained by checking whether the protein is annotated in a certain GO term or KEGG pathway. The linkage of proteins and drug compounds can be retrieved from STITCH (Search Tool for Interactions of Chemicals, http://stitch.embl.de/) [18], a large-scale source providing associations between chemicals and between chemicals and proteins. These associations include both known and predicted associations. Chemicals and proteins are linked according to evidence gathered through experiments, databases or the literature. The information that is provided by STITCH has been used to investigate various compound-related problems [6,19–24]. In the obtained file (protein_chemical.links.detailed.v4.0.tsv.gz), each association contained one chemical and one protein and scores measuring the strength of the association from different aspects. Here, we focused on whether a given chemical and a given protein occur in the file as an association. This information was used to refine the investigated dataset (see Section 2.1) and encode each drug compound in *S* (see Section 2.3).

### Encoding method

To indicate the association between drug compounds and GO terms or KEGG pathways, we employed the enrichment theory of GO terms and KEGG pathways to represent each drug compound. For a certain drug compound *d*, let *G*(*d*) be a protein set containing human proteins that have associations with *d* that can be easily obtained using the information that is mentioned in Section 2.2.

#### GO enrichment

Given one drug *d* and one GO term GO_*j*_, the GO enrichment score is defined as the—log_10_ of the hypergeometric test *P* value [25–27] of *G*(*d*) and GO term GO_*j*_, which can be calculated by

$${\text{S}}_{\text{GO}}\left(d,{\text{GO}}_{j}\right)=-{\text{log}}_{10}(\sum_{k=m}^{n}\frac{\left(\begin{array}{c} M\\ m \end{array}\right)\;\left(\begin{array}{c} N-M\\ n-m \end{array}\right)}{\left(\begin{array}{c} N\\ n \end{array}\right)})$$ (1)

where *N*, *M*, *n* and *m* are the total number of proteins in humans, the number of proteins that are annotated to the GO term GO_*j*_, the number of proteins in *G*(*d*), and the number of proteins both in *G*(*d*) and annotated to the GO term GO_*j*_, respectively. If the GO enrichment score is high for one drug and one GO term, they have a strong association. A total of 17,904 GO terms were adopted to extract 17,904 GO enrichment scores.

#### KEGG enrichment

Similar to the definition of the GO enrichment score, given as one drug *d* and one KEGG pathway P_*j*_, the KEGG enrichment score [27] is defined as follows:

$${\text{S}}_{\text{KEGG}}\;\left(d,{\text{P}}_{j}\right)=-{\text{log}}_{10}(\sum_{k=m}^{n}\frac{\left(\begin{array}{c} M\\ m \end{array}\right)\left(\begin{array}{c} N-M\\ n-m \end{array}\right)}{\left(\begin{array}{c} N\\ n \end{array}\right)})$$ (2)

where the meanings of *N* and *n* are same as those in **Eq 1**, and *M* and *m* are the number of proteins in the KEGG pathway P_*j*_ and the number of proteins both in *G*(*d*) and P_*j*_, respectively. Similarly, drug *d* and pathway P_*j*_ have a strong association if the KEGG enrichment score between them is high. A total of 279 KEGG pathways were used to extract 279 KEGG enrichment scores.

It can be observed from the above two paragraphs that the number of features in GO terms was much larger than that in KEGG pathways. To fairly analyze the contribution of GO terms and KEGG pathways, we constructed two datasets, *S*_KEGG_ and *S*_GO_, from *S*, where each sample in *S*_KEGG_ was represented by 279 KEGG enrichment scores, and each sample in *S*_GO_ was represented by 17,904 GO enrichment scores.

### mRMR

As described in Section 2.3, each drug was represent by 279 features of enrichment scores in the KEGG pathway or 17,904 GO enrichment scores. These scores indicate the associations between drugs and their corresponding GO terms or KEGG pathways. However, not all GO terms or KEGG pathways play the same role in the determination of drug target-based classes. Some of these terms and pathways may indicate key contributions, while others have few associations. To analyze these features (*i*.*e*., GO terms and KEGG pathways), a popular feature selection method (mRMR) was employed. This method was first proposed by Peng *et al*. [17] and to date has been used to analyze various complicated biological systems [28–35] because it has two excellent criteria: Max-Relevance and Min-Redundancy. One of the main outputs of the mRMR program is the MaxRel feature list, in which features are sorted based on their contribution to the classification. The detailed procedure is as follows: Let *x* be a variable representing the samples’ class labels and *y* be another variable representing the values of all samples under a certain feature. Then, the association between the samples’ class labels and the feature can be measured by the mutual information (MI) of *x* and *y* as computed by

$$I(x,y)=\iint p(x,y)\text{log}\frac{p(x,y)}{p(x)p(y)}dxdy$$ (3)

where *p*(*x*) and *p*(*y*) denote the marginal probabilities of *x* and *y*, respectively, and *p*(*x*, *y*) denotes the joint probabilistic distribution of *x* and *y*. MI is considered an ideal stochastic dependence measurement [36], as it can detect not only linear but also non-linear dependencies and can capture the heterogeneity of association [37]. The MaxRel feature list sorts features according to the values as calculated by **Eq 3**, in that features with high values as calculated by **Eq 3**would receive high places in the MaxRel feature list.

## Results and Discussion

### Results of mRMR method

The mRMR method was used to analyze the GO terms and KEGG pathways (http://research.janelia.org/peng/proj/mRMR/). For convenience, it was executed with default parameters on the datasets *S*_KEGG_ and *S*_GO_. As a result, we obtained two MaxRel feature lists that sorted features from the KEGG pathways and GO terms according to the values as calculated by **Eq 3**. These two lists are available in **S2** and **S3 Tables**, respectively, although the list of GO terms only includes the first 500 GO term features due to the computational time. Additionally, the MI value for each listed feature is also available in **S2**and **S3 Tables**. Because features with high MI values have strong associations for the determination of drug target-based classes, we selected 19 features from KEGG pathways with MI values larger than or equal to 0.05 and 45 GO term features with MI values greater than or equal to 0.1. These KEGG pathways and GO terms are termed hereafter as key KEGG pathways and key GO terms.

### Mean value of the key KEGG pathways and GO terms for each class

**Fig 1:**
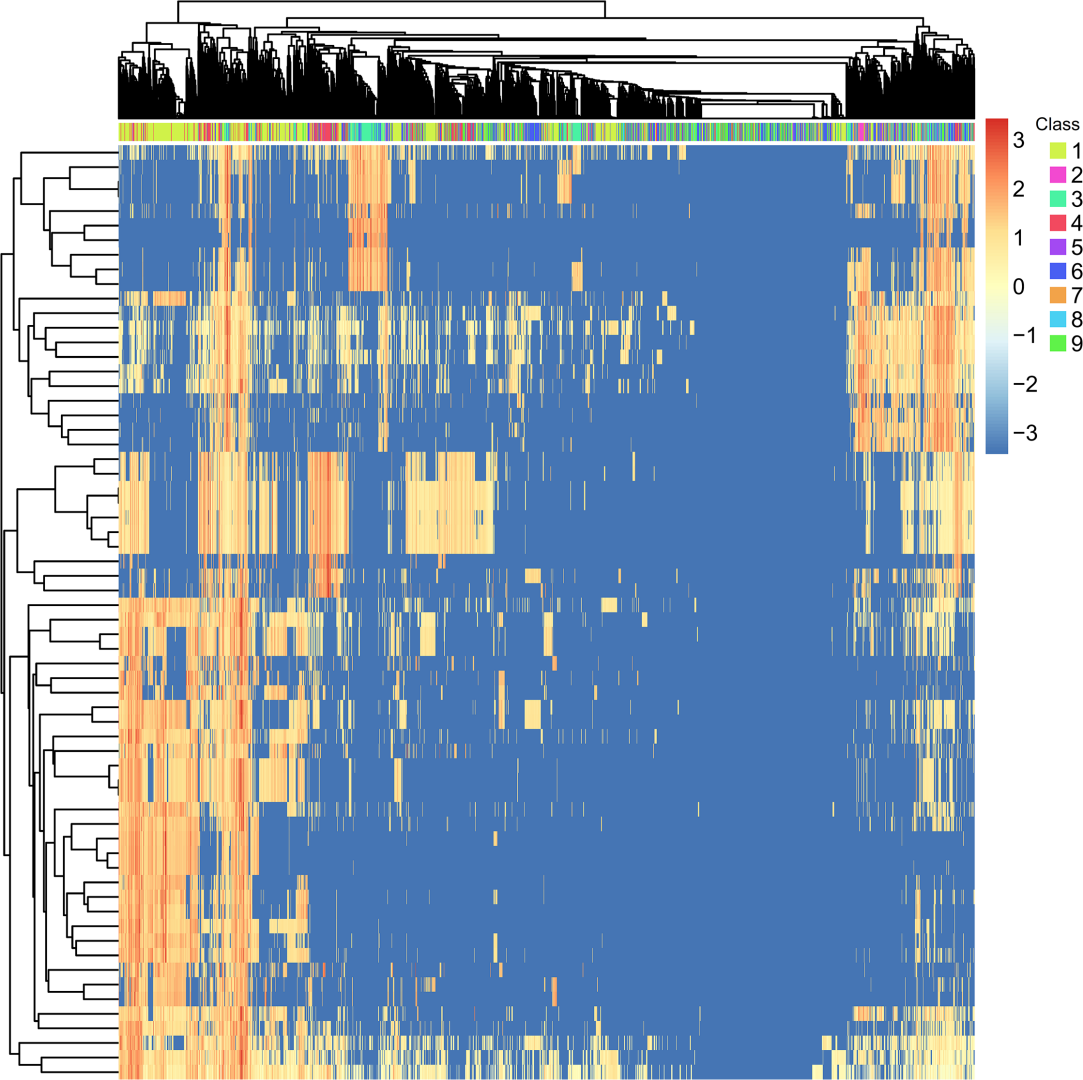
The heat map of the enrichment scores of all 2,015 drug compounds on key KEGG pathways and GO terms. In the heat map, rows are KEGG pathways and GO terms, and columns are drugs. The drug classes are the same as in **Table 1**. The matrix is row-wise normalized, and warmer colors represent higher enrichment scores. On the left side, there is a cluster corresponding to GPCR, but other small clusters are not very clear.

In **Fig 1**, we plotted the enrichment scores of all 2,015 drug compounds on key KEGG pathways and GO terms. On the left side, there was a cluster corresponding to GPCR, but other small clusters were not very clear. It was difficult to analyze the key KEGG pathways and GO terms based solely on their enrichment scores for drug compounds, as each class contained multiple drug compounds. Therefore, it was necessary to refine their values as follows: For each key KEGG pathway and one target-based class, we calculated the level value, which was defined as the average of the enrichment scores under this KEGG pathway for all of the drug compounds in this class. Similarly, we defined the level value of each key GO term and each target-based class. The level values of nine target-based classes on the key KEGG pathways and GO terms can be found in **S4 Table**. In addition to the level values of nine classes and the MI value, we also calculated the traditional Analysis of variance (ANOVA) p value. The ANOVA p values in nine out of 19 KEGG pathways and 40 out of 45 GO terms were smaller than 0.05. Both the MI and ANOVA results suggested that the enrichment scores of key KEGG pathways and GO terms were significantly different among different classes of drugs.

**Fig 2:**
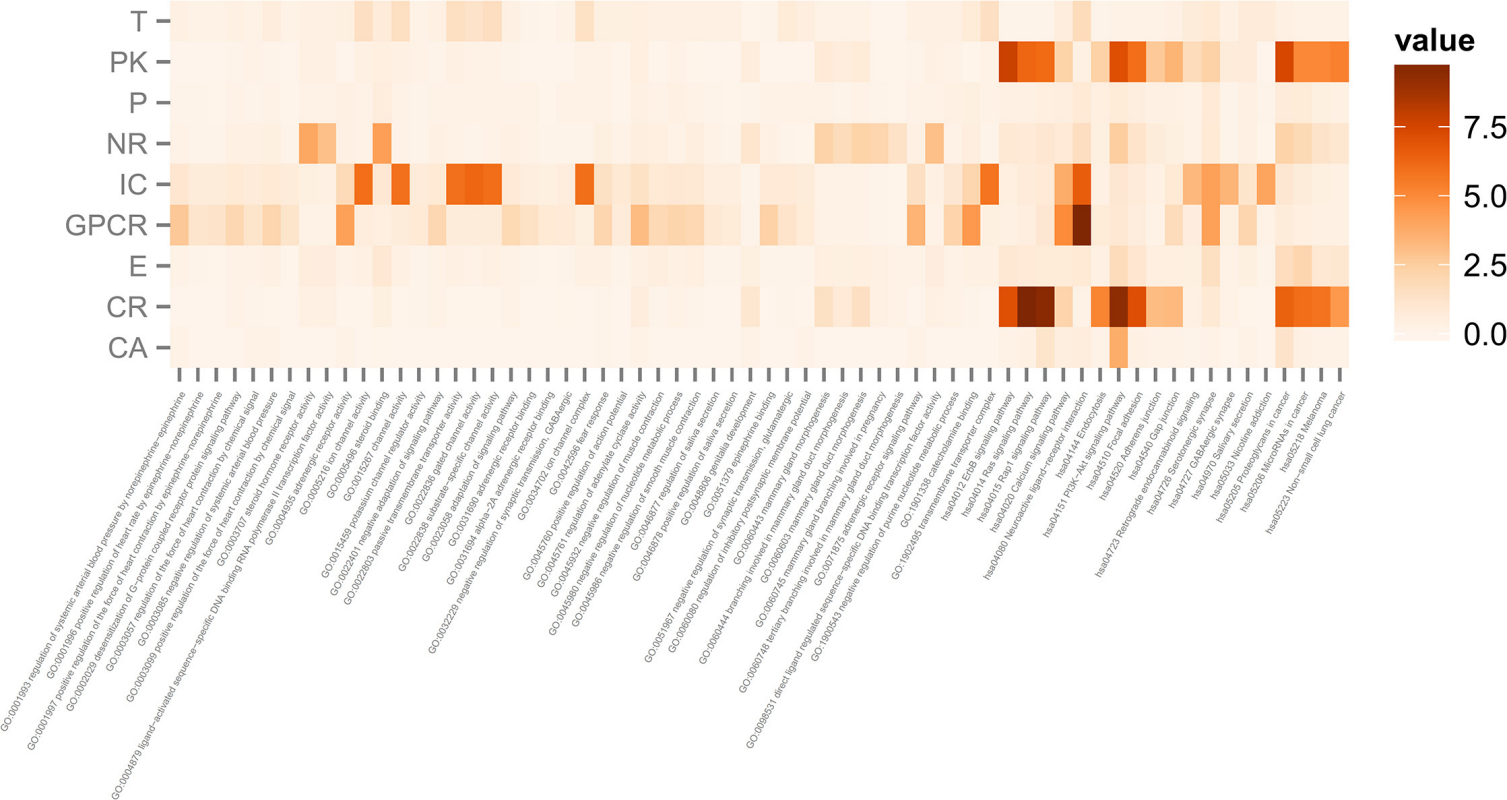
The heat map of the level values of each target-based class on key KEGG pathways and GO terms. The rows are drug classes, and the columns are KEGG pathways and GO terms. Darker colors represent higher mean values, *i*.*e*., average enrichment scores.

For certain key KEGG pathways or GO terms, the high level value of one target-based class indicated that the drugs in this class may have high enrichment, thereby implying that this feature may provide key contributions for the identification of drugs in this class from other drugs. To clearly show the mean value for different target-based classes for certain key KEGG pathways or GO terms, we plotted a heat map for the key KEGG pathways or GO terms, as shown in **Fig 2**. The following sections provide a detailed discussion of **Fig 2**.

### Different level values of the GO and KEGG enrichment of nine drug categories

KEGG DRUG provides a drug information resource based on chemical structures and classifies drugs into nine categories based on their targets. In this study, to better understand the mechanisms of existing drugs and provide clues for drug interaction and the future prediction of DTIs, we associated drug targets with biological functions by analyzing the distribution of both 2,015 drugs and their nine categories in 19 KEGG pathways and 45 GO terms. The nine drug categories show different enrichment levels in GO terms and KEGG pathways, implying the diversity in the biological function enrichment of each drug category.

Specifically, the GPCR category included 657 drug compounds that target G protein-coupled receptors (GPCRs). GPCRs are seven-transmembrane domain receptors and constitute a large protein family that binds to signaling molecules outside the cell and activates signal transduction pathways and cellular responses inside the cell. GPCRs are common drug targets and were estimated to serve as targets of approximately 40% of modern medical drugs [38]. Based on our analysis, class 1 drugs were highly enriched in the hsa04080 “neuroactive ligand-receptor interaction pathway” with a level value 9.88. The hsa04080 (neuroactive ligand-receptor interaction) pathway contains many GPCRs, including growth hormone secretagogue receptor (GHSR), gonadotropin-releasing hormone receptor (GNRHR), leucine-rich repeat-containing G protein-coupled receptor 7/8 (LGR7/8), corticotrophin-releasing hormone receptor 1/2 (CRHR1/2), gastrin-releasing peptide receptor (GRPR), neuromedin U receptor 1/2 (NMUR1/2) and tachykinin receptor 1/2/3 (TACR1/2/3), indicating the indispensable function of GPCR signaling in neuronal cells [39,40].

Similarly, the CR category included 35 drug compounds that target cytokine receptors (CRs). CRs are a family of either membrane-bound or soluble receptors that binds cytokines and can be classified into several subfamilies. The drugs in the CR category were highly enriched in the hsa04014 “Ras signaling pathway” (level value = 9.89), hsa04015 “Rap1 signaling pathway” (level value = 9.54) and hsa04151 “PI3K-Akt signaling pathway” (level value = 9.37). These results suggest that these drugs tend to act on the same pathway. The cell surface CRs (EGFR, FGFR1/2/3/4, NGFR, insulin receptor (INSR) and IGF1R) play crucial roles in signaling transduction. Ras and Ras-like small GTPase Rap1 are upstream of many protein kinases, including Raf1 AKT and PIK3C. Rap1 signaling functions in integrin activation, cell shape determination, and adherens junction formation [41]. Furthermore, for the PI3K-Akt signaling pathway, CRs, including EGFR, FGFR1/2/3/4, NGFR, and INSR and PK proteins such as AKT, MAP2K1/2, and PDPK1, are involved in this pathway.

Comparatively, drugs that target transporters (Ts) and pathogens (Ps) do not have highly enriched functions. Ts are a family of membrane proteins that are involved in the movement of ions, small molecules or macromolecules to cross a biological membrane [42]. Ps include a wide range of infectious agents, such as a virus, bacterium, prion, fungus or protozoan [43]. Their top enriched functions are hsa04080 neuroactive ligand-receptor interaction, but the level values are low (1.75 and 0.87). These results suggest that although these drugs share the same class of targets, they vary in biological functions due to different enriched pathways.

### Potential application of our method in drug interaction and DTI prediction

Our analysis revealed enriched GO and KEGG pathways of nine drug categories. Among these pathways, some GO terms or KEGG pathways are highly enriched by several drug categories. For example, hsa04080 neuroactive ligand-receptor interaction pathway was enriched by GPCR (level value = 9.88) and IC (level value = 6.62) category drugs, and the hsa04151 PI3K-Akt signaling pathway was enriched by CR (level value = 9.37) and PK (level value = 7.10) category drugs. PI3K-Akt signaling pathways are crucial to many aspects of cell growth and survival under both physiological and pathological conditions, such as cancer [44]. These results indicate that although many drugs have different targets, they are involved in the same biological pathway and are likely to have potential synergistic drug interactions.

For DTI prediction, two major methods are extensively used: the traditional drug discovery method, in which new drugs are predicted for a certain target, and the chemical biology method, in which new potential targets are predicted for a given drug [45]. Here, our analysis not only provides the overall distribution of each drug category for KEGG pathways and GO terms but also provides a reference to each drug. This information can help predict new DTIs.

## Conclusion

This study analyzed a drug target-based classification system using the enrichment theory of gene ontology and the KEGG pathway. The minimum redundancy maximum relevance method was used to analyze the contribution of each GO term and KEGG pathway to determine drug target-based classes. The analysis results suggest that some GO terms and KEGG pathways are important for the identification of drug target-based classes. We hope that these findings promote the comprehension of this classification system and the study of drug-target interactions.

## Supporting Information

S1 Table The codes of 2,015 drug compounds and their target-based classes. (PDF)

S2 Table The MaxRel feature list for the features about KEGG pathways. (PDF)

S3 Table The MaxRel feature list for the features about GO terms. (PDF)

S4 Table The level values of nine target-based classes, MI values and ANOVA p values on the 19 key KEGG pathways and 45 key GO terms. (XLSX)
