# Supplementary material for: Gene Ontology and KEGG Pathway Enrichment Analysis of a Drug Target-Based Classification System
Source: PLoS One. 2015 May 7;10(5):e0126492. doi: 10.1371/journal.pone.0126492 (PMC4423955; doi:10.1371/journal.pone.0126492)
Supplement: S1 Table — (PDF) [file pone.0126492.s001.pdf]

S1 Table. The codes of 2,015 drug compounds and their target-based classes.

**Compound ID**	**Target-based Class**
CID000000237	E
CID000000338	E
CID000000359	E
CID000000564	E
CID000000681	GPCR
CID000000750	IC
CID000000774	GPCR
CID000000785	E
CID000000896	GPCR
CID000000951	GPCR
CID000001046	P
CID000001234	IC
CID000001613	E
CID000001676	GPCR
CID000001775	IC
CID000001783	P
CID000001935	E
CID000001967	E
CID000001978	GPCR
CID000001979	GPCR

CID000001981	E
CID000001983	E
CID000001986	E
CID000001990	E
CID000001993	GPCR
CID000002083	GPCR
CID000002088	E
CID000002092	GPCR
CID000002097	E
CID000002099	GPCR
CID000002118	IC
CID000002119	GPCR
CID000002120	E
CID000002130	P
CID000002131	E
CID000002132	IC
CID000002137	IC
CID000002145	E
CID000002160	IC
CID000002162	IC
CID000002163	IC
CID000002165	E

CID000002169	GPCR
CID000002170	IC
CID000002176	E
CID000002179	E
CID000002187	E
CID000002200	GPCR
CID000002216	GPCR
CID000002218	IC
CID000002225	IC
CID000002239	GPCR
CID000002244	E
CID000002247	GPCR
CID000002249	GPCR
CID000002264	GPCR
CID000002266	P
CID000002267	GPCR
CID000002273	T
CID000002284	GPCR
CID000002294	IC
CID000002309	GPCR
CID000002315	T
CID000002327	E

CID000002337	IC
CID000002343	T
CID000002351	IC
CID000002366	GPCR
CID000002369	GPCR
CID000002370	GPCR
CID000002372	GPCR
CID000002375	NR
CID000002378	P
CID000002381	GPCR
CID000002405	GPCR
CID000002431	IC
CID000002435	GPCR
CID000002441	IC
CID000002448	GPCR
CID000002449	GPCR
CID000002465	GPCR
CID000002468	PK
CID000002471	T
CID000002472	GPCR
CID000002473	GPCR
CID000002474	IC

CID000002475	GPCR
CID000002477	GPCR
CID000002482	IC
CID000002484	P
CID000002520	IC
CID000002540	GPCR
CID000002541	GPCR
CID000002551	GPCR
CID000002554	IC
CID000002564	GPCR
CID000002577	E
CID000002581	E
CID000002583	GPCR
CID000002585	GPCR
CID000002662	E
CID000002663	GPCR
CID000002678	GPCR
CID000002712	IC
CID000002720	T
CID000002725	GPCR
CID000002726	GPCR
CID000002727	IC

CID000002732	T
CID000002747	IC
CID000002750	NR
CID000002754	E
CID000002755	GPCR
CID000002756	GPCR
CID000002762	P
CID000002763	NR
CID000002764	P
CID000002769	GPCR
CID000002771	IC
CID000002780	GPCR
CID000002783	GPCR
CID000002789	IC
CID000002796	NR
CID000002798	IC
CID000002801	IC
CID000002802	IC
CID000002803	GPCR
CID000002809	IC
CID000002811	IC
CID000002812	P

CID000002816	IC
CID000002818	GPCR
CID000002880	P
CID000002905	GPCR
CID000002913	GPCR
CID000002955	P
CID000002995	IC
CID000002997	IC
CID000002998	IC
CID000003007	IC
CID000003009	E
CID000003016	IC
CID000003019	IC
CID000003025	IC
CID000003032	E
CID000003038	E
CID000003039	E
CID000003042	GPCR
CID000003059	E
CID000003089	GPCR
CID000003100	GPCR
CID000003103	GPCR

CID000003105	GPCR
CID000003108	E
CID000003114	IC
CID000003117	E
CID000003148	GPCR
CID000003151	GPCR
CID000003152	E
CID000003157	GPCR
CID000003162	GPCR
CID000003168	GPCR
CID000003180	IC
CID000003191	GPCR
CID000003198	P
CID000003202	E
CID000003219	GPCR
CID000003229	P
CID000003241	GPCR
CID000003261	IC
CID000003278	T
CID000003290	GPCR
CID000003291	IC
CID000003295	E

CID000003306	GPCR
CID000003307	IC
CID000003308	E
CID000003324	P
CID000003333	IC
CID000003339	NR
CID000003340	GPCR
CID000003342	E
CID000003343	GPCR
CID000003345	GPCR
CID000003348	GPCR
CID000003354	E
CID000003355	IC
CID000003357	P
CID000003360	E
CID000003365	P
CID000003369	IC
CID000003371	E
CID000003372	GPCR
CID000003380	IC
CID000003385	E
CID000003386	IC

CID000003388	GPCR
CID000003389	GPCR
CID000003393	IC
CID000003394	E
CID000003395	E
CID000003396	IC
CID000003397	NR
CID000003400	IC
CID000003406	E
CID000003410	GPCR
CID000003414	P
CID000003436	E
CID000003446	IC
CID000003447	E
CID000003448	IC
CID000003450	IC
CID000003454	P
CID000003463	NR
CID000003467	P
CID000003475	IC
CID000003476	IC
CID000003478	IC

CID000003487	IC
CID000003488	IC
CID000003494	GPCR
CID000003519	GPCR
CID000003547	PK
CID000003559	GPCR
CID000003563	IC
CID000003604	IC
CID000003608	IC
CID000003609	GPCR
CID000003627	GPCR
CID000003639	T
CID000003647	T
CID000003655	E
CID000003657	E
CID000003658	GPCR
CID000003671	GPCR
CID000003672	E
CID000003673	E
CID000003675	E
CID000003676	IC
CID000003696	IC

CID000003698	E
CID000003699	E
CID000003702	T
CID000003715	E
CID000003748	E
CID000003749	GPCR
CID000003759	E
CID000003760	P
CID000003762	GPCR
CID000003775	GPCR
CID000003779	GPCR
CID000003783	GPCR
CID000003784	IC
CID000003792	E
CID000003821	IC
CID000003822	GPCR
CID000003825	E
CID000003826	E
CID000003827	GPCR
CID000003869	GPCR
CID000003878	IC
CID000003883	E

CID000003899	E
CID000003902	E
CID000003911	GPCR
CID000003914	GPCR
CID000003926	IC
CID000003932	IC
CID000003936	P
CID000003947	IC
CID000003948	P
CID000003949	IC
CID000003957	GPCR
CID000003958	IC
CID000003961	GPCR
CID000003963	P
CID000003964	GPCR
CID000003965	E
CID000003969	GPCR
CID000003995	GPCR
CID000004004	E
CID000004008	IC
CID000004011	IC
CID000004020	IC

CID000004032	IC
CID000004034	GPCR
CID000004036	E
CID000004041	IC
CID000004044	E
CID000004047	T
CID000004051	E
CID000004054	IC
CID000004057	GPCR
CID000004058	GPCR
CID000004060	IC
CID000004062	IC
CID000004064	IC
CID000004066	GPCR
CID000004086	GPCR
CID000004091	PK
CID000004097	GPCR
CID000004099	IC
CID000004100	E
CID000004121	T
CID000004134	GPCR
CID000004167	GPCR

CID000004168	GPCR
CID000004170	T
CID000004171	GPCR
CID000004174	E
CID000004177	IC
CID000004178	IC
CID000004184	GPCR
CID000004189	P
CID000004192	IC
CID000004195	GPCR
CID000004197	E
CID000004201	IC
CID000004205	GPCR
CID000004211	E
CID000004212	E
CID000004235	E
CID000004237	E
CID000004249	GPCR
CID000004257	GPCR
CID000004259	P
CID000004260	GPCR
CID000004261	E

CID000004380	E
CID000004409	E
CID000004416	NR
CID000004417	GPCR
CID000004418	GPCR
CID000004421	P
CID000004436	GPCR
CID000004440	GPCR
CID000004449	GPCR
CID000004454	P
CID000004456	E
CID000004463	P
CID000004472	E
CID000004473	IC
CID000004485	IC
CID000004486	IC
CID000004493	NR
CID000004494	IC
CID000004495	E
CID000004496	IC
CID000004497	IC
CID000004499	IC

CID000004500	IC
CID000004506	IC
CID000004507	IC
CID000004528	IC
CID000004539	P
CID000004543	IC
CID000004567	GPCR
CID000004583	P
CID000004585	GPCR
CID000004593	E
CID000004594	E
CID000004595	GPCR
CID000004601	GPCR
CID000004613	GPCR
CID000004614	E
CID000004628	P
CID000004631	GPCR
CID000004634	GPCR
CID000004636	GPCR
CID000004641	E
CID000004642	GPCR
CID000004644	E

CID000004673	E
CID000004679	E
CID000004680	E
CID000004688	E
CID000004725	P
CID000004726	T
CID000004737	IC
CID000004747	GPCR
CID000004748	GPCR
CID000004760	E
CID000004761	GPCR
CID000004763	IC
CID000004768	GPCR
CID000004781	E
CID000004820	IC
CID000004822	GPCR
CID000004823	E
CID000004826	IC
CID000004828	GPCR
CID000004829	NR
CID000004830	GPCR
CID000004831	P

CID000004839	GPCR
CID000004841	GPCR
CID000004843	T
CID000004845	GPCR
CID000004848	GPCR
CID000004849	T
CID000004870	T
CID000004887	GPCR
CID000004888	E
CID000004890	IC
CID000004893	GPCR
CID000004906	IC
CID000004913	IC
CID000004914	IC
CID000004916	GPCR
CID000004917	GPCR
CID000004919	GPCR
CID000004922	GPCR
CID000004926	GPCR
CID000004927	GPCR
CID000004932	IC
CID000004934	GPCR

CID000004935	IC
CID000004942	GPCR
CID000004943	IC
CID000004946	GPCR
CID000004991	E
CID000004992	GPCR
CID000004993	P
CID000004999	IC
CID000005002	GPCR
CID000005029	E
CID000005035	NR
CID000005069	IC
CID000005071	P
CID000005073	GPCR
CID000005074	GPCR
CID000005078	GPCR
CID000005090	E
CID000005095	GPCR
CID000005105	GPCR
CID000005147	E
CID000005152	GPCR
CID000005160	GPCR

CID000005161	E
CID000005193	IC
CID000005205	GPCR
CID000005210	IC
CID000005212	E
CID000005214	P
CID000005215	P
CID000005233	E
CID000005245	E
CID000005265	GPCR
CID000005278	E
CID000005311	E
CID000005314	IC
CID000005318	P
CID000005320	P
CID000005323	P
CID000005328	P
CID000005329	P
CID000005333	P
CID000005335	P
CID000005343	P
CID000005344	P

CID000005355	GPCR
CID000005357	GPCR
CID000005358	GPCR
CID000005360	IC
CID000005374	GPCR
CID000005379	P
CID000005386	E
CID000005391	IC
CID000005401	GPCR
CID000005403	GPCR
CID000005405	GPCR
CID000005411	IC
CID000005419	GPCR
CID000005452	GPCR
CID000005457	GPCR
CID000005467	GPCR
CID000005468	E
CID000005472	GPCR
CID000005473	GPCR
CID000005474	GPCR
CID000005476	GPCR
CID000005482	P

CID000005487	GPCR
CID000005503	IC
CID000005504	GPCR
CID000005505	IC
CID000005506	P
CID000005507	E
CID000005508	E
CID000005510	P
CID000005517	P
CID000005524	GPCR
CID000005526	E
CID000005531	E
CID000005546	IC
CID000005556	IC
CID000005560	T
CID000005566	GPCR
CID000005567	GPCR
CID000005568	GPCR
CID000005572	GPCR
CID000005573	GPCR
CID000005574	GPCR
CID000005578	P

CID000005582	E
CID000005584	IC
CID000005587	GPCR
CID000005591	NR
CID000005593	GPCR
CID000005596	GPCR
CID000005606	GPCR
CID000005625	P
CID000005639	GPCR
CID000005648	GPCR
CID000005656	IC
CID000005663	E
CID000005665	E
CID000005666	IC
CID000005707	GPCR
CID000005709	GPCR
CID000005717	GPCR
CID000005720	E
CID000005724	E
CID000005732	IC
CID000005734	IC
CID000005735	IC

CID000005736	GPCR
CID000005743	NR
CID000005744	NR
CID000005745	NR
CID000005754	NR
CID000005755	NR
CID000005756	NR
CID000005757	NR
CID000005768	IC
CID000005770	T
CID000005773	P
CID000005775	GPCR
CID000005790	E
CID000005803	NR
CID000005815	GPCR
CID000005819	NR
CID000005825	IC
CID000005832	NR
CID000005833	NR
CID000005834	NR
CID000005853	E
CID000005857	NR

CID000005858	NR
CID000005865	NR
CID000005870	NR
CID000005876	NR
CID000005877	NR
CID000005878	NR
CID000005881	NR
CID000005904	P
CID000005905	P
CID000005906	GPCR
CID000005909	GPCR
CID000005917	IC
CID000005920	NR
CID000005936	E
CID000005952	NR
CID000005959	P
CID000005965	E
CID000005983	E
CID000005991	NR
CID000005992	E
CID000005994	NR
CID000005995	NR

CID000006000	IC
CID000006005	GPCR
CID000006010	NR
CID000006013	NR
CID000006018	T
CID000006024	P
CID000006032	P
CID000006041	GPCR
CID000006047	GPCR
CID000006081	GPCR
CID000006087	P
CID000006098	P
CID000006166	NR
CID000006169	GPCR
CID000006196	P
CID000006215	NR
CID000006216	NR
CID000006230	NR
CID000006234	P
CID000006238	NR
CID000006249	P
CID000006252	E

CID000006256	P
CID000006279	NR
CID000006292	IC
CID000006300	NR
CID000006307	T
CID000006436	NR
CID000006446	NR
CID000006468	IC
CID000006662	P
CID000006719	E
CID000006726	GPCR
CID000006741	NR
CID000006832	GPCR
CID000006834	GPCR
CID000006869	P
CID000007028	GPCR
CID000007172	GPCR
CID000007534	GPCR
CID000008223	GPCR
CID000008225	NR
CID000008226	GPCR
CID000008229	GPCR

CID000008230	GPCR
CID000008249	PK
CID000008271	IC
CID000008366	E
CID000008378	P
CID000008612	IC
CID000008982	P
CID000009051	NR
CID000009270	NR
CID000009294	GPCR
CID000009324	NR
CID000009400	NR
CID000009403	NR
CID000009416	NR
CID000009417	GPCR
CID000009429	GPCR
CID000009444	E
CID000009564	IC
CID000009565	IC
CID000009642	NR
CID000009651	E
CID000009677	NR

CID000009681	GPCR
CID000009782	NR
CID000009864	E
CID000009878	NR
CID000009880	NR
CID000009904	NR
CID000010101	IC
CID000010114	E
CID000010180	E
CID000010255	IC
CID000010517	GPCR
CID000010531	GPCR
CID000010547	E
CID000010631	NR
CID000010635	NR
CID000010660	GPCR
CID000010836	IC
CID000011151	E
CID000011259	IC
CID000011290	GPCR
CID000011683	NR
CID000012555	E

CID000012559	P
CID000012560	P
CID000013109	NR
CID000013308	NR
CID000013313	NR
CID000013314	IC
CID000013765	NR
CID000013769	E
CID000013789	NR
CID000013791	NR
CID000014051	P
CID000014687	NR
CID000014743	NR
CID000015020	NR
CID000015130	GPCR
CID000015209	NR
CID000015541	P
CID000016078	GPCR
CID000016106	GPCR
CID000016229	GPCR
CID000016230	IC
CID000016362	GPCR

CID000016490	NR
CID000016533	NR
CID000016623	NR
CID000016752	NR
CID000016923	NR
CID000016960	GPCR
CID000017134	P
CID000017925	IC
CID000018026	GPCR
CID000018283	P
CID000018343	E
CID000018381	P
CID000019003	P
CID000019063	E
CID000019090	NR
CID000019135	NR
CID000019143	GPCR
CID000019150	P
CID000019648	P
CID000019861	GPCR
CID000020298	GPCR
CID000020313	IC

CID000020469	NR
CID000020824	P
CID000021115	NR
CID000021138	GPCR
CID000021319	P
CID000021453	P
CID000021653	P
CID000021700	NR
CID000021704	P
CID000021800	NR
CID000021826	NR
CID000021846	GPCR
CID000021855	GPCR
CID000022502	P
CID000023087	P
CID000023897	GPCR
CID000024066	P
CID000024199	GPCR
CID000024904	IC
CID000025050	E
CID000025096	GPCR
CID000025103	GPCR

CID000025249	NR
CID000025654	GPCR
CID000026133	NR
CID000026388	GPCR
CID000026533	IC
CID000026757	E
CID000027200	P
CID000027350	IC
CID000027400	GPCR
CID000027447	P
CID000027881	E
CID000027991	GPCR
CID000028417	NR
CID000028620	E
CID000028693	GPCR
CID000028864	GPCR
CID000029029	P
CID000029435	E
CID000030131	GPCR
CID000030323	E
CID000030667	GPCR
CID000030699	P

CID000030842	GPCR
CID000031100	GPCR
CID000031113	E
CID000031307	NR
CID000031378	NR
CID000031477	GPCR
CID000031703	E
CID000031728	GPCR
CID000032169	IC
CID000032778	IC
CID000032798	NR
CID000033036	GPCR
CID000033039	P
CID000033255	P
CID000033477	P
CID000033613	P
CID000033624	GPCR
CID000033625	GPCR
CID000033630	IC
CID000033649	IC
CID000033672	P
CID000033741	GPCR

CID000034040	GPCR
CID000034041	E
CID000034312	IC
CID000034521	E
CID000034632	IC
CID000034768	P
CID000035329	GPCR
CID000035370	P
CID000036119	P
CID000036143	E
CID000036273	P
CID000036283	GPCR
CID000036294	P
CID000036462	E
CID000036523	GPCR
CID000036709	NR
CID000036811	GPCR
CID000036920	GPCR
CID000036921	P
CID000036975	GPCR
CID000037264	GPCR
CID000037460	GPCR

CID000037464	GPCR
CID000037497	IC
CID000037542	P
CID000037768	P
CID000038077	CA
CID000038103	P
CID000038521	GPCR
CID000038945	IC
CID000039031	P
CID000039042	NR
CID000039147	GPCR
CID000039186	IC
CID000039468	GPCR
CID000039764	IC
CID000039912	E
CID000039941	E
CID000040240	P
CID000040973	NR
CID000041049	GPCR
CID000041376	GPCR
CID000041386	T
CID000041693	GPCR

CID000041774	E
CID000041781	T
CID000041835	GPCR
CID000041867	E
CID000042008	P
CID000042368	E
CID000042396	GPCR
CID000042890	E
CID000043157	E
CID000043507	P
CID000043593	P
CID000043672	P
CID000043708	P
CID000043815	IC
CID000044093	E
CID000044112	GPCR
CID000044187	P
CID000044219	E
CID000044570	E
CID000044601	GPCR
CID000047320	IC
CID000047471	P

CID000047499	P
CID000047528	IC
CID000047725	GPCR
CID000047811	GPCR
CID000047964	P
CID000048040	IC
CID000050138	NR
CID000050192	IC
CID000050224	GPCR
CID000050287	GPCR
CID000050515	E
CID000050599	P
CID000050893	GPCR
CID000051038	P
CID000051044	GPCR
CID000051081	P
CID000051173	E
CID000051263	GPCR
CID000051550	GPCR
CID000051565	P
CID000051634	E
CID000051710	GPCR

CID000052542	E
CID000052919	GPCR
CID000053000	E
CID000053024	P
CID000053232	E
CID000053275	IC
CID000053359	E
CID000053388	GPCR
CID000053555	E
CID000053568	E
CID000053697	GPCR
CID000053708	E
CID000054259	P
CID000054331	P
CID000054343	GPCR
CID000054384	GPCR
CID000054454	E
CID000054477	GPCR
CID000054562	GPCR
CID000054687	E
CID000054746	GPCR
CID000054747	E

CID000054765	GPCR
CID000054797	E
CID000054840	IC
CID000054889	E
CID000054891	E
CID000054897	IC
CID000054949	E
CID000055185	P
CID000055190	GPCR
CID000055245	NR
CID000055256	P
CID000055283	P
CID000055390	E
CID000055472	GPCR
CID000055480	IC
CID000055483	GPCR
CID000055651	GPCR
CID000055891	E
CID000055917	E
CID000056031	GPCR
CID000056068	NR
CID000056207	P

CID000056237	E
CID000056329	E
CID000056338	IC
CID000056801	GPCR
CID000056971	GPCR
CID000057030	E
CID000057109	GPCR
CID000057242	GPCR
CID000057363	E
CID000057469	CA
CID000058504	IC
CID000058573	E
CID000059226	GPCR
CID000059691	GPCR
CID000059693	E
CID000059768	GPCR
CID000059798	IC
CID000060020	P
CID000060062	P
CID000060149	GPCR
CID000060164	NR
CID000060168	IC

CID000060172	P
CID000060198	E
CID000060464	P
CID000060490	E
CID000060496	IC
CID000060510	IC
CID000060539	GPCR
CID000060558	GPCR
CID000060561	E
CID000060594	E
CID000060606	GPCR
CID000060648	IC
CID000060652	E
CID000060656	GPCR
CID000060662	IC
CID000060691	E
CID000060692	E
CID000060697	GPCR
CID000060699	E
CID000060713	GPCR
CID000060734	P
CID000060737	CA

CID000060746	P
CID000060749	E
CID000060752	IC
CID000060769	GPCR
CID000060774	GPCR
CID000060781	E
CID000060786	P
CID000060788	E
CID000060789	GPCR
CID000060795	GPCR
CID000060809	GPCR
CID000060814	GPCR
CID000060816	E
CID000060819	GPCR
CID000060822	E
CID000060824	E
CID000060825	P
CID000060834	IC
CID000060837	E
CID000060846	GPCR
CID000060852	E
CID000060853	GPCR

CID000060855	P
CID000060857	GPCR
CID000060870	NR
CID000060871	P
CID000060876	P
CID000060877	P
CID000060910	NR
CID000060934	P
CID000060949	GPCR
CID000060953	E
CID000060955	E
CID000062115	P
CID000062156	GPCR
CID000062819	GPCR
CID000062873	GPCR
CID000062886	IC
CID000062956	E
CID000062959	P
CID000063009	IC
CID000063019	NR
CID000063043	NR
CID000063044	NR

CID000063046	NR
CID000063047	NR
CID000064139	P
CID000064142	P
CID000064147	P
CID000064627	GPCR
CID000064642	IC
CID000064644	IC
CID000064706	P
CID000064710	P
CID000064718	E
CID000064725	P
CID000064782	GPCR
CID000064922	E
CID000065013	P
CID000065014	CR
CID000065016	P
CID000065028	P
CID000065157	NR
CID000065435	GPCR
CID000065449	IC
CID000065478	NR

CID000065498	GPCR
CID000065501	IC
CID000065598	E
CID000065646	P
CID000065755	P
CID000065772	GPCR
CID000065814	GPCR
CID000065825	IC
CID000065856	IC
CID000065864	P
CID000065866	IC
CID000065881	E
CID000065914	IC
CID000065922	GPCR
CID000065941	IC
CID000065948	IC
CID000065957	P
CID000065981	IC
CID000065998	IC
CID000065999	GPCR
CID000066249	NR
CID000066264	GPCR

CID000066440	NR
CID000068555	GPCR
CID000068582	NR
CID000068601	GPCR
CID000068613	GPCR
CID000068634	GPCR
CID000068682	P
CID000068712	GPCR
CID000068736	IC
CID000068740	E
CID000068741	E
CID000068770	GPCR
CID000068783	NR
CID000068802	E
CID000068844	E
CID000068861	NR
CID000068873	NR
CID000068942	E
CID000070881	E
CID000071141	P
CID000071231	IC
CID000071301	GPCR

CID000071307	E
CID000071310	GPCR
CID000071320	E
CID000071329	IC
CID000071339	P
CID000071348	GPCR
CID000071355	E
CID000071360	GPCR
CID000071371	NR
CID000071392	P
CID000071414	NR
CID000071446	P
CID000071469	P
CID000071534	GPCR
CID000071616	P
CID000071640	GPCR
CID000071698	GPCR
CID000071734	E
CID000071739	GPCR
CID000071763	GPCR
CID000071809	GPCR
CID000071906	NR

CID000071926	E
CID000071955	GPCR
CID000071961	P
CID000071986	GPCR
CID000071992	E
CID000072004	E
CID000072006	GPCR
CID000072014	GPCR
CID000072015	P
CID000072036	GPCR
CID000072059	E
CID000072171	E
CID000072231	CA
CID000072286	GPCR
CID000072396	P
CID000072474	P
CID000072492	P
CID000072981	P
CID000073011	P
CID000073115	P
CID000073303	P
CID000073491	P

CID000077990	E
CID000077992	GPCR
CID000077993	GPCR
CID000077997	GPCR
CID000077998	NR
CID000082146	NR
CID000082148	GPCR
CID000082153	NR
CID000083786	E
CID000083898	GPCR
CID000084003	E
CID000084019	GPCR
CID000084029	P
CID000091276	GPCR
CID000091438	NR
CID000091499	GPCR
CID000091562	P
CID000091610	IC
CID000091668	NR
CID000092253	IC
CID000092727	P
CID000093184	P

CID000093504	IC
CID000102146	NR
CID000102232	NR
CID000104741	NR
CID000104758	E
CID000104762	E
CID000104838	P
CID000104849	GPCR
CID000104865	GPCR
CID000107688	E
CID000107706	E
CID000107807	E
CID000107930	GPCR
CID000107969	GPCR
CID000107994	E
CID000107999	GPCR
CID000108182	GPCR
CID000110634	E
CID000110635	E
CID000114897	E
CID000114905	GPCR
CID000115162	P

CID000115355	E
CID000115367	GPCR
CID000119031	E
CID000119170	IC
CID000119182	E
CID000119259	IC
CID000119369	GPCR
CID000119569	GPCR
CID000119583	GPCR
CID000119607	E
CID000119828	E
CID000121888	GPCR
CID000121891	IC
CID000121892	IC
CID000121903	GPCR
CID000123600	GPCR
CID000123606	GPCR
CID000123607	GPCR
CID000123619	E
CID000123630	P
CID000123631	CR
CID000123879	GPCR

CID000124087	GPCR
CID000124088	P
CID000124093	P
CID000125017	IC
CID000125564	GPCR
CID000125846	P
CID000126941	E
CID000127527	P
CID000129211	GPCR
CID000129319	GPCR
CID000130313	P
CID000130564	E
CID000130881	GPCR
CID000130918	GPCR
CID000130956	E
CID000131535	P
CID000131601	E
CID000131682	E
CID000132519	E
CID000132706	GPCR
CID000132790	GPCR
CID000132916	GPCR

CID000133016	GPCR
CID000133078	GPCR
CID000133081	GPCR
CID000133371	GPCR
CID000133621	GPCR
CID000134018	E
CID000146294	GPCR
CID000146570	IC
CID000148192	P
CID000148195	E
CID000148200	IC
CID000148202	E
CID000149096	P
CID000150171	GPCR
CID000150311	T
CID000150610	P
CID000151166	E
CID000151170	E
CID000151171	GPCR
CID000151172	T
CID000151193	CR
CID000153241	E

CID000153270	E
CID000153751	E
CID000153941	P
CID000153994	IC
CID000153997	E
CID000154256	NR
CID000154413	NR
CID000154417	GPCR
CID000154575	E
CID000155435	NR
CID000155773	GPCR
CID000155815	E
CID000156321	GPCR
CID000156328	IC
CID000156333	GPCR
CID000156345	P
CID000156391	E
CID000156413	CR
CID000156418	GPCR
CID000156422	PK
CID000156689	GPCR
CID000157049	E

CID000157838	E
CID000157918	GPCR
CID000158781	GPCR
CID000158783	GPCR
CID000158793	GPCR
CID000158795	GPCR
CID000159269	P
CID000159271	E
CID000159324	E
CID000159594	GPCR
CID000160024	E
CID000162302	GPCR
CID000162581	NR
CID000162834	GPCR
CID000162862	P
CID000163296	P
CID000163838	IC
CID000164509	IC
CID000164521	GPCR
CID000164739	GPCR
CID000165579	P
CID000166543	GPCR

CID000166553	E
CID000166558	E
CID000166562	E
CID000167441	P
CID000169075	P
CID000169870	NR
CID000170361	IC
CID000171758	P
CID000171808	P
CID000171883	P
CID000172306	GPCR
CID000172997	GPCR
CID000174174	GPCR
CID000175540	GPCR
CID000175804	IC
CID000176166	PK
CID000177335	GPCR
CID000178029	GPCR
CID000178052	IC
CID000178265	P
CID000179337	NR
CID000179344	IC

CID000180081	IC
CID000181625	NR
CID000183797	E
CID000183811	GPCR
CID000189729	E
CID000189821	NR
CID000190816	E
CID000193962	P
CID000198125	E
CID000202225	P
CID000204101	E
CID000204105	IC
CID000204106	E
CID000204109	NR
CID000206044	NR
CID000208898	IC
CID000208902	GPCR
CID000208907	E
CID000208908	CR
CID000208910	E
CID000208928	GPCR
CID000208951	GPCR

CID000213039	P
CID000213043	P
CID000213046	GPCR
CID000214356	P
CID000216210	E
CID000216235	GPCR
CID000216416	NR
CID000216456	IC
CID000216466	GPCR
CID000219024	GPCR
CID000219100	E
CID000219101	IC
CID000222757	NR
CID000222786	NR
CID000225609	NR
CID000229021	NR
CID000229295	NR
CID000229455	NR
CID000235905	NR
CID000240767	NR
CID000247839	NR
CID000251636	NR

CID000252372	NR
CID000294641	E
CID000337359	E
CID000383413	GPCR
CID000387447	E
CID000392622	P
CID000439260	GPCR
CID000439285	GPCR
CID000439299	E
CID000439302	GPCR
CID000439501	E
CID000439693	E
CID000440707	NR
CID000441129	P
CID000441199	P
CID000441207	E
CID000441233	E
CID000441277	GPCR
CID000441290	IC
CID000441300	P
CID000441313	E
CID000441314	E

CID000441335	NR
CID000441336	NR
CID000441341	T
CID000441350	E
CID000441371	P
CID000441383	P
CID000441397	P
CID000441401	P
CID000441404	NR
CID000441406	NR
CID000443371	GPCR
CID000443372	GPCR
CID000443375	GPCR
CID000443376	E
CID000443380	E
CID000443382	P
CID000443387	P
CID000443408	GPCR
CID000443868	IC
CID000443872	NR
CID000443873	E
CID000443884	GPCR

CID000443889	GPCR
CID000443894	GPCR
CID000443935	NR
CID000443947	NR
CID000443951	GPCR
CID000443953	P
CID000443958	NR
CID000443980	NR
CID000444008	NR
CID000444020	E
CID000444021	P
CID000444025	NR
CID000444028	E
CID000444030	GPCR
CID000444036	NR
CID000444499	E
CID000445643	E
CID000446156	E
CID000446157	E
CID000446541	E
CID000446556	E
CID000446596	P

CID000446727	P
CID000447715	NR
CID000448537	NR
CID000449171	NR
CID000449193	E
CID000449459	NR
CID000451447	P
CID000451668	E
CID000452191	E
CID000456255	P
CID000464205	P
CID000466151	E
CID000467825	P
CID000477468	P
CID000604519	E
CID000636374	NR
CID000636380	E
CID000636402	E
CID000636405	P
CID000636411	E
CID000638678	GPCR
CID000644025	IC

CID000644073	GPCR
CID000656511	P
CID000656575	P
CID000656580	P
CID000656583	E
CID000656615	NR
CID000656664	GPCR
CID000656667	IC
CID000656804	NR
CID000657201	GPCR
CID000657298	E
CID000657308	GPCR
CID000667476	NR
CID000667493	E
CID000667550	E
CID000688272	GPCR
CID000941361	IC
CID000941651	GPCR
CID000969472	IC
CID001349907	E
CID001547484	GPCR
CID001548886	E

CID001548943	IC
CID001548952	NR
CID001548954	NR
CID001548971	E
CID001548992	GPCR
CID001548999	GPCR
CID001549007	P
CID001549119	E
CID002724385	E
CID002733509	E
CID002733525	NR
CID002826718	P
CID003000226	P
CID003000715	IC
CID003001028	NR
CID003001055	GPCR
CID003001322	CR
CID003002820	P
CID003005572	NR
CID003009355	CR
CID003032285	IC
CID003032325	NR

CID003033151	GPCR
CID003033538	GPCR
CID003033637	GPCR
CID003033676	GPCR
CID003033702	E
CID003033766	NR
CID003033825	E
CID003033860	IC
CID003033887	GPCR
CID003033968	NR
CID003033988	P
CID003034010	E
CID003034011	NR
CID003035240	GPCR
CID003036505	NR
CID003037705	NR
CID003038497	IC
CID003038522	CR
CID003042089	GPCR
CID003052762	GPCR
CID003052775	E
CID003055168	NR

CID003058754	E
CID003081361	CR
CID003081912	GPCR
CID003082555	GPCR
CID003083544	GPCR
CID003086400	GPCR
CID003086651	E
CID003086671	E
CID004369359	E
CID004375468	GPCR
CID004659569	E
CID005001396	E
CID005280360	GPCR
CID005280363	GPCR
CID005280427	GPCR
CID005280453	NR
CID005280723	GPCR
CID005280754	E
CID005280793	NR
CID005280795	NR
CID005280980	P
CID005281004	NR

CID005281006	P
CID005281034	NR
CID005281037	GPCR
CID005281040	GPCR
CID005281042	IC
CID005281074	GPCR
CID005281075	GPCR
CID005281078	E
CID005281081	E
CID005281107	GPCR
CID005281881	GPCR
CID005282136	GPCR
CID005282138	IC
CID005282165	P
CID005282175	GPCR
CID005282181	NR
CID005282183	E
CID005282188	P
CID005282190	NR
CID005282192	P
CID005282205	GPCR
CID005282211	P

CID005282226	GPCR
CID005282229	NR
CID005282234	GPCR
CID005282241	P
CID005282375	NR
CID005282381	GPCR
CID005282415	GPCR
CID005282425	IC
CID005282432	P
CID005282437	P
CID005282439	E
CID005282442	GPCR
CID005282449	GPCR
CID005282451	E
CID005282592	GPCR
CID005282593	GPCR
CID005284371	GPCR
CID005284402	E
CID005284514	GPCR
CID005284527	P
CID005284533	NR
CID005284537	NR

CID005284543	GPCR
CID005284549	E
CID005284554	P
CID005284555	NR
CID005284566	GPCR
CID005284569	GPCR
CID005284570	GPCR
CID005284592	GPCR
CID005284594	GPCR
CID005284595	GPCR
CID005284596	GPCR
CID005284599	GPCR
CID005284603	GPCR
CID005284604	GPCR
CID005284612	NR
CID005284627	IC
CID005287541	E
CID005287969	E
CID005288320	E
CID005288826	GPCR
CID005289223	GPCR
CID005311027	GPCR

CID005311035	GPCR
CID005311037	GPCR
CID005311051	NR
CID005311064	GPCR
CID005311066	NR
CID005311067	NR
CID005311068	GPCR
CID005311101	NR
CID005311181	GPCR
CID005311217	IC
CID005311221	GPCR
CID005311225	GPCR
CID005311236	GPCR
CID005311304	GPCR
CID005311309	IC
CID005311398	IC
CID005311412	NR
CID005311413	GPCR
CID005311424	GPCR
CID005311447	E
CID005311505	NR
CID005311507	GPCR

CID005312125	GPCR
CID005312153	GPCR
CID005324345	IC
CID005328940	PK
CID005329098	CR
CID005352062	E
CID005353853	P
CID005353894	E
CID005359271	GPCR
CID005359272	GPCR
CID005359371	GPCR
CID005359476	E
CID005360515	GPCR
CID005360696	IC
CID005360697	IC
CID005361092	GPCR
CID005361880	GPCR
CID005361917	GPCR
CID005362065	P
CID005362114	P
CID005362115	E
CID005362118	E

CID005362123	E
CID005362129	E
CID005362417	E
CID005362422	E
CID005362436	GPCR
CID005362440	P
CID005381226	P
CID005388961	E
CID005462328	GPCR
CID005462501	E
CID005463984	E
CID005464105	E
CID005464201	E
CID005464343	E
CID005464355	P
CID005475158	GPCR
CID005479529	P
CID005479530	P
CID005479537	P
CID005479539	P
CID005480431	P
CID005481173	P

CID005481350	P
CID005484727	E
CID005486182	P
CID005486198	IC
CID005486684	GPCR
CID005486971	IC
CID005487888	P
CID005488547	GPCR
CID005489436	GPCR
CID005490674	E
CID005491954	P
CID005493444	E
CID005497196	PK
CID005702062	GPCR
CID005702160	GPCR
CID005742673	P
CID005742832	P
CID005743186	P
CID006321416	P
CID006323490	P
CID006323497	P
CID006324663	GPCR

CID006328657	P
CID006335986	P
CID006336505	P
CID006337614	GPCR
CID006347538	E
CID006400678	P
CID006410758	P
CID006433090	GPCR
CID006433095	GPCR
CID006433110	T
CID006433122	GPCR
CID006435415	E
CID006436055	P
CID006436090	E
CID006436123	GPCR
CID006436135	GPCR
CID006436168	P
CID006436640	GPCR
CID006437850	E
CID006437877	P
CID006438339	GPCR
CID006438378	GPCR

CID006439420	E
CID006444692	CR
CID006445230	GPCR
CID006445562	CR
CID006450173	CA
CID006450333	GPCR
CID006450546	IC
CID006450800	E
CID006450815	GPCR
CID006450822	GPCR
CID006451325	E
CID006456014	E
CID006506050	GPCR
CID006509849	GPCR
CID006509979	E
CID006526396	P
CID006533629	P
CID006537431	P
CID006540466	P
CID006540478	NR
CID006604100	IC
CID006708482	E

CID006713928	P
CID006714002	NR
CID006914163	T
CID006915944	P
CID006917715	NR
CID006917778	IC
CID006917815	E
CID006917951	GPCR
CID006917956	E
CID006918029	GPCR
CID006918091	E
CID006918106	GPCR
CID006918107	GPCR
CID006918130	E
CID006918140	GPCR
CID006918141	NR
CID006918155	NR
CID006918191	E
CID006918218	P
CID006918236	IC
CID006918248	GPCR
CID006918266	GPCR

CID006918278	E
CID006918296	E
CID006918305	IC
CID006918330	GPCR
CID006918336	E
CID006918365	GPCR
CID006918366	GPCR
CID006918387	GPCR
CID006918456	GPCR
CID006918486	E
CID006918493	GPCR
CID006918523	E
CID006918524	GPCR
CID006918548	NR
CID006918554	GPCR
CID006918558	GPCR
CID006918580	GPCR
CID006918602	E
CID006918638	E
CID006918837	E
CID009568614	E
CID009570757	P

CID009571001	GPCR
CID009571003	IC
CID009571107	P
CID009574101	E
CID009577221	NR
CID009578005	E
CID009578243	E
CID009578572	E
CID009795739	GPCR
CID009796068	E
CID009796181	E
CID009796590	E
CID009804204	GPCR
CID009805430	GPCR
CID009818306	NR
CID009820073	E
CID009821849	E
CID009821951	GPCR
CID009823820	CR
CID009824145	IC
CID009824350	E
CID009825285	GPCR

CID009826744	GPCR
CID009828911	GPCR
CID009830519	P
CID009832383	GPCR
CID009837243	T
CID009838021	GPCR
CID009838712	CR
CID009838762	GPCR
CID009843089	E
CID009844194	GPCR
CID009851821	E
CID009854011	GPCR
CID009854489	NR
CID009857255	GPCR
CID009859832	E
CID009862248	NR
CID009865515	E
CID009865528	GPCR
CID009865554	CR
CID009867642	GPCR
CID009868491	GPCR
CID009869929	E

CID009871074	PK
CID009871419	GPCR
CID009871420	T
CID009875401	E
CID009881504	IC
CID009883933	IC
CID009884366	GPCR
CID009886917	E
CID009887712	T
CID009887754	E
CID009887812	GPCR
CID009888484	NR
CID009892480	GPCR
CID009892860	E
CID009910098	GPCR
CID009911830	CR
CID009912771	E
CID009913767	GPCR
CID009913881	E
CID009915743	CR
CID009918079	GPCR
CID009924495	IC

CID009926791	PK
CID009931953	E
CID009933475	CR
CID009939609	E
CID009949848	NR
CID009952884	E
CID009954033	NR
CID009961434	E
CID009966051	GPCR
CID009977819	CR
CID009980241	NR
CID009997821	P
CID010041070	NR
CID010052040	GPCR
CID010071196	GPCR
CID010077129	GPCR
CID010095005	NR
CID010096344	E
CID010102486	IC
CID010113978	CR
CID010116877	GPCR
CID010117987	GPCR

CID010127622	PK
CID010152654	E
CID010182969	E
CID010184653	CR
CID010184665	GPCR
CID010216431	E
CID010220503	GPCR
CID010250769	P
CID010274777	NR
CID010275777	E
CID010280735	E
CID010286159	NR
CID010295295	NR
CID010296883	PK
CID010302451	PK
CID010343641	GPCR
CID010345213	GPCR
CID010360683	NR
CID010366136	CR
CID010440987	GPCR
CID010453870	T
CID010459564	GPCR

CID010461508	PK
CID011154555	GPCR
CID011154925	CR
CID011167602	CR
CID011188409	E
CID011204019	P
CID011223423	GPCR
CID011226090	GPCR
CID011234052	CR
CID011238823	GPCR
CID011243969	E
CID011250647	GPCR
CID011253490	E
CID011281011	GPCR
CID011285791	CR
CID011319053	GPCR
CID011340891	GPCR
CID011347535	E
CID011395145	NR
CID011411233	P
CID011417567	E
CID011430856	GPCR

CID011452716	CA
CID011467166	GPCR
CID011494412	PK
CID011504294	GPCR
CID011516136	E
CID011519069	GPCR
CID011526696	GPCR
CID011552706	PK
CID011556711	E
CID011561674	E
CID011597537	PK
CID011597697	IC
CID011604525	GPCR
CID011626560	CR
CID011634458	E
CID011634973	GPCR
CID011640390	CR
CID011641515	E
CID011658655	IC
CID011658859	GPCR
CID011667893	CR
CID011671467	PK

CID011683556	GPCR
CID011707110	PK
CID011749858	E
CID011876263	NR
CID011949652	E
CID011954223	GPCR
CID011954290	IC
CID011954310	NR
CID011960529	E
CID011979316	GPCR
CID011988953	T
CID012358971	IC
CID015052414	NR
CID015547703	E
CID015940185	P
CID015942715	NR
CID015983988	E
CID016004692	GPCR
CID016058810	T
CID016066663	GPCR
CID016070111	CA
CID016071605	GPCR

CID016088021	E
CID016129616	GPCR
CID016129617	GPCR
CID016129620	GPCR
CID016129672	CR
CID016129681	GPCR
CID016129682	GPCR
CID016129690	IC
CID016129703	E
CID016129704	E
CID016130938	GPCR
CID016130957	GPCR
CID016131215	GPCR
CID016132283	GPCR
CID016132357	GPCR
CID016132392	GPCR
CID016132418	CR
CID016132438	CR
CID016132441	E
CID016136245	GPCR
CID016157882	GPCR
CID016197723	CA

CID016220188	PK
CID016394563	E
CID016678941	T
CID016683012	E
CID016683028	NR
CID020055008	NR
CID020629114	GPCR
CID021309251	P
CID021874557	GPCR
CID023083982	P
CID023652731	E
CID023653789	GPCR
CID023668479	P
CID023690938	E
CID023724523	P
CID023724530	PK
CID023724531	PK
CID023724781	GPCR
CID023724842	P
CID023724872	P
CID023724885	P
CID023724902	GPCR

CID023725064	GPCR
CID023725083	P
CID023725088	GPCR
CID023725625	E
CID023727689	GPCR
CID024771867	PK
CID024775005	GPCR
CID024776445	GPCR
CID024795088	NR
CID024812758	T
CID024826799	PK
CID024838347	GPCR
CID024847745	P
CID024889392	CR
CID025070031	GPCR
CID025074469	GPCR
CID025074886	GPCR
CID025077649	GPCR
CID025102847	CR
CID025126797	PK
CID025183872	E
CID025195354	GPCR

CID025201794	E
CID026275995	P
CID027885548	P
CID042611257	PK
CID044146714	CR
CID044201125	E
CID044462760	PK
CID044513473	E
CID046908928	T
CID046926350	PK
CID049787020	P
CID051508717	E
CID051529148	E
CID051537605	P
CID051600099	P
CID051601240	P
CID051655231	P
CID053297329	P
CID053297362	GPCR
CID053325981	E
CID053487898	E
CID054671203	P

CID054675768	P
CID054675779	P
CID054675785	P
CID054676537	E
CID054680692	E
CID054684141	E
CID054685524	E
CID054686183	P
CID054686350	E
CID054686904	P
CID054690031	E
CID054698175	E
CID054708299	E
CID056603661	P
CID056841789	CA
