# Supplementary material for: Gene Ontology and KEGG Pathway Enrichment Analysis of a Drug Target-Based Classification System
Source: PLoS One. 2015 May 7;10(5):e0126492. doi: 10.1371/journal.pone.0126492 (PMC4423955; doi:10.1371/journal.pone.0126492)
Supplement: S2 Table — (PDF) [file pone.0126492.s002.pdf]

S2 Table. The MaxRel feature list for the features about KEGG pathways.

Rank	Feature name	Score
1	hsa04970	0.084
2	hsa04080	0.075
3	hsa04540	0.071
4	hsa04020	0.071
5	hsa05033	0.065
6	hsa04727	0.064
7	hsa04723	0.062
8	hsa05206	0.061
9	hsa04510	0.06
10	hsa04726	0.06
11	hsa04012	0.06
12	hsa04144	0.059
13	hsa04520	0.058
14	hsa04015	0.057
15	hsa04014	0.055
16	hsa04151	0.054
17	hsa05223	0.054
18	hsa05218	0.053
19	hsa05205	0.051
20	hsa04261	0.049

21	hsa05160	0.048
22	hsa05200	0.048
23	hsa05215	0.047
24	hsa05219	0.046
25	hsa05221	0.046
26	hsa04062	0.045
27	hsa04010	0.045
28	hsa04370	0.044
29	hsa04728	0.044
30	hsa04722	0.043
31	hsa05213	0.043
32	hsa04917	0.043
33	hsa04725	0.042
34	hsa05220	0.041
35	hsa05212	0.041
36	hsa04960	0.04
37	hsa04060	0.04
38	hsa04810	0.04
39	hsa04068	0.039
40	hsa05214	0.039
41	hsa05340	0.039
42	hsa04961	0.038

43	hsa04650	0.038
44	hsa04911	0.038
45	hsa05162	0.038
46	hsa05202	0.037
47	hsa04270	0.037
48	hsa04066	0.037
49	hsa04360	0.036
50	hsa05120	0.036
51	hsa04750	0.036
52	hsa00240	0.035
53	hsa04930	0.035
54	hsa04660	0.035
55	hsa04919	0.035
56	hsa04320	0.034
57	hsa05169	0.034
58	hsa04150	0.034
59	hsa04914	0.033
60	hsa05031	0.033
61	hsa04664	0.033
62	hsa05412	0.033
63	hsa05216	0.032
64	hsa03018	0.032

65	hsa04910	0.032
66	hsa05032	0.032
67	hsa04260	0.031
68	hsa04912	0.03
69	hsa05161	0.03
70	hsa04713	0.029
71	hsa04670	0.029
72	hsa04380	0.029
73	hsa05030	0.029
74	hsa03020	0.029
75	hsa05130	0.029
76	hsa05131	0.028
77	hsa05414	0.028
78	hsa05211	0.028
79	hsa04720	0.027
80	hsa04110	0.026
81	hsa04114	0.026
82	hsa05168	0.026
83	hsa04530	0.026
84	hsa04921	0.026
85	hsa04064	0.026
86	hsa03320	0.026

87	hsa05164	0.026
88	hsa04666	0.025
89	hsa04978	0.025
90	hsa05145	0.025
91	hsa05012	0.024
92	hsa04724	0.024
93	hsa00910	0.024
94	hsa00590	0.024
95	hsa04913	0.024
96	hsa04971	0.024
97	hsa00630	0.024
98	hsa05100	0.024
99	hsa04662	0.024
100	hsa04920	0.023
101	hsa04620	0.023
102	hsa05210	0.023
103	hsa04974	0.023
104	hsa04115	0.022
105	hsa00591	0.022
106	hsa04630	0.022
107	hsa00140	0.022
108	hsa05222	0.022

109	hsa04614	0.021
110	hsa04623	0.021
111	hsa04977	0.021
112	hsa04730	0.021
113	hsa00790	0.021
114	hsa04964	0.021
115	hsa05203	0.021
116	hsa05323	0.021
117	hsa00670	0.021
118	hsa05144	0.02
119	hsa04622	0.02
120	hsa03420	0.02
121	hsa05166	0.02
122	hsa05132	0.02
123	hsa04742	0.02
124	hsa04744	0.02
125	hsa03460	0.02
126	hsa04610	0.019
127	hsa03440	0.019
128	hsa00561	0.019
129	hsa04966	0.019
130	hsa04350	0.019

131	hsa05034	0.019
132	hsa05410	0.019
133	hsa04973	0.018
134	hsa00650	0.018
135	hsa00531	0.018
136	hsa00520	0.018
137	hsa04140	0.018
138	hsa05142	0.018
139	hsa05020	0.017
140	hsa04975	0.017
141	hsa04916	0.017
142	hsa04668	0.017
143	hsa05134	0.017
144	hsa05143	0.017
145	hsa04640	0.017
146	hsa04740	0.017
147	hsa04310	0.016
148	hsa01040	0.016
149	hsa05416	0.016
150	hsa04340	0.016
151	hsa04721	0.016
152	hsa05152	0.016

153	hsa05146	0.016
154	hsa00232	0.016
155	hsa04611	0.016
156	hsa04210	0.016
157	hsa05140	0.016
158	hsa00120	0.016
159	hsa02010	0.015
160	hsa04142	0.015
161	hsa04621	0.015
162	hsa04932	0.015
163	hsa03060	0.015
164	hsa00982	0.015
165	hsa00533	0.015
166	hsa00592	0.014
167	hsa00190	0.014
168	hsa04120	0.014
169	hsa00230	0.014
170	hsa05110	0.014
171	hsa04145	0.014
172	hsa00983	0.014
173	hsa00830	0.013
174	hsa00980	0.013

175	hsa00061	0.013
176	hsa04710	0.013
177	hsa05321	0.013
178	hsa05204	0.013
179	hsa03430	0.013
180	hsa00330	0.013
181	hsa05010	0.013
182	hsa05016	0.013
183	hsa03410	0.013
184	hsa04146	0.012
185	hsa03022	0.012
186	hsa00740	0.012
187	hsa00770	0.012
188	hsa00562	0.012
189	hsa05320	0.012
190	hsa05217	0.012
191	hsa00460	0.012
192	hsa03013	0.012
193	hsa00280	0.012
194	hsa00052	0.012
195	hsa04390	0.012
196	hsa05332	0.012

197	hsa00620	0.012
198	hsa00340	0.011
199	hsa00512	0.011
200	hsa04915	0.011
201	hsa04070	0.011
202	hsa04972	0.011
203	hsa00030	0.011
204	hsa00040	0.011
205	hsa00380	0.011
206	hsa03010	0.011
207	hsa00130	0.01
208	hsa04976	0.01
209	hsa04330	0.01
210	hsa00400	0.01
211	hsa05150	0.01
212	hsa00072	0.01
213	hsa04514	0.01
214	hsa04612	0.01
215	hsa04940	0.01
216	hsa03008	0.01
217	hsa05133	0.01
218	hsa00920	0.01

219	hsa00785	0.01
220	hsa05014	0.01
221	hsa00270	0.01
222	hsa00010	0.01
223	hsa04141	0.01
224	hsa00511	0.009
225	hsa04512	0.009
226	hsa03450	0.009
227	hsa05330	0.009
228	hsa03030	0.009
229	hsa00970	0.009
230	hsa00480	0.009
231	hsa05310	0.009
232	hsa00860	0.009
233	hsa05322	0.009
234	hsa00051	0.009
235	hsa00310	0.009
236	hsa00640	0.008
237	hsa00053	0.008
238	hsa04918	0.008
239	hsa04122	0.008
240	hsa04962	0.008

241	hsa04130	0.008
242	hsa00900	0.007
243	hsa00250	0.007
244	hsa00430	0.007
245	hsa00514	0.007
246	hsa00260	0.007
247	hsa00600	0.007
248	hsa00410	0.007
249	hsa00500	0.007
250	hsa03050	0.006
251	hsa00601	0.006
252	hsa00564	0.006
253	hsa00062	0.006
254	hsa04672	0.006
255	hsa00471	0.005
256	hsa00534	0.005
257	hsa00565	0.005
258	hsa00071	0.005
259	hsa00360	0.005
260	hsa00760	0.004
261	hsa04950	0.004
262	hsa00780	0.004

263	hsa00350	0.004
264	hsa00510	0.004
265	hsa03040	0.004
266	hsa00750	0.004
267	hsa00100	0.004
268	hsa00532	0.003
269	hsa00563	0.003
270	hsa00450	0.003
271	hsa03015	0.002
272	hsa00020	0.002
273	hsa00524	0.002
274	hsa00604	0.002
275	hsa00730	0.002
276	hsa00300	0.002
277	hsa00603	0.001
278	hsa00290	0.001
