# Supplementary material for: Gene Ontology and KEGG Pathway Enrichment Analysis of a Drug Target-Based Classification System
Source: PLoS One. 2015 May 7;10(5):e0126492. doi: 10.1371/journal.pone.0126492 (PMC4423955; doi:10.1371/journal.pone.0126492)
Supplement: S3 Table — (PDF) [file pone.0126492.s003.pdf]

S3 Table. The MaxRel feature list for the features about GO terms.

Rank	Feature name	Score
1	GO:0034702	0.138
2	GO:0060603	0.136
3	GO:0060745	0.133
4	GO:0004935	0.131
5	GO:0001993	0.131
6	GO:0001997	0.13
7	GO:1902495	0.129
8	GO:0031694	0.128
9	GO:0003707	0.128
10	GO:0071875	0.128
11	GO:0005496	0.127
12	GO:0003099	0.127
13	GO:0031690	0.125
14	GO:0045986	0.125
15	GO:0045932	0.124
16	GO:0060443	0.123
17	GO:0046877	0.122
18	GO:0051379	0.119
19	GO:0005216	0.119
20	GO:1901338	0.117

21	GO:0003085	0.117
22	GO:0022838	0.115
23	GO:0001996	0.112
24	GO:0015267	0.112
25	GO:0022803	0.112
26	GO:0060444	0.111
27	GO:0022836	0.111
28	GO:0051967	0.111
29	GO:0015459	0.11
30	GO:0060080	0.109
31	GO:0098531	0.107
32	GO:0004879	0.107
33	GO:0060748	0.105
34	GO:0022401	0.104
35	GO:0023058	0.104
36	GO:0002029	0.104
37	GO:0045761	0.104
38	GO:0042596	0.102
39	GO:0045760	0.101
40	GO:0045980	0.1
41	GO:1900543	0.1
42	GO:0003057	0.1

43	GO:0046878	0.1
44	GO:0048806	0.1
45	GO:0032229	0.1
46	GO:0060740	0.099
47	GO:0030800	0.099
48	GO:0001965	0.099
49	GO:0045744	0.098
50	GO:0007193	0.098
51	GO:0004939	0.098
52	GO:0002025	0.098
53	GO:0030815	0.098
54	GO:0030518	0.097
55	GO:0060512	0.097
56	GO:0002024	0.097
57	GO:0008179	0.097
58	GO:0007210	0.096
59	GO:0030850	0.096
60	GO:0048149	0.095
61	GO:0051380	0.095
62	GO:0050951	0.095
63	GO:0051481	0.094
64	GO:0061377	0.094

65	GO:0060749	0.094
66	GO:0060742	0.094
67	GO:0060736	0.094
68	GO:0004993	0.094
69	GO:0040015	0.093
70	GO:0050432	0.093
71	GO:0003056	0.093
72	GO:0031280	0.092
73	GO:1901020	0.092
74	GO:0014059	0.092
75	GO:0014046	0.092
76	GO:0002031	0.092
77	GO:0008227	0.092
78	GO:0030520	0.092
79	GO:0050433	0.092
80	GO:0007191	0.091
81	GO:0030809	0.091
82	GO:1900372	0.091
83	GO:0030803	0.091
84	GO:0045776	0.091
85	GO:0004883	0.091
86	GO:0051350	0.091

87	GO:0060359	0.091
88	GO:0007194	0.091
89	GO:0007268	0.091
90	GO:0043950	0.09
91	GO:0030818	0.09
92	GO:0035809	0.09
93	GO:0005980	0.09
94	GO:0009251	0.09
95	GO:0098900	0.09
96	GO:0051378	0.09
97	GO:0048148	0.09
98	GO:0051937	0.09
99	GO:0042220	0.089
100	GO:0022612	0.089
101	GO:0003321	0.089
102	GO:0035811	0.089
103	GO:0015075	0.088
104	GO:0043176	0.088
105	GO:0002021	0.088
106	GO:0014848	0.087
107	GO:0006367	0.087
108	GO:0045762	0.086

109	GO:0060081	0.086
110	GO:0015872	0.086
111	GO:0051952	0.086
112	GO:0006939	0.086
113	GO:0060687	0.086
114	GO:0006936	0.086
115	GO:0060599	0.085
116	GO:0051339	0.085
117	GO:0010460	0.085
118	GO:0046541	0.085
119	GO:0007198	0.085
120	GO:0006352	0.085
121	GO:0022891	0.085
122	GO:0031946	0.084
123	GO:0060158	0.084
124	GO:0044247	0.084
125	GO:0043567	0.084
126	GO:0022857	0.084
127	GO:0051583	0.084
128	GO:0051934	0.084
129	GO:0090493	0.084
130	GO:0090494	0.084

131	GO:0014832	0.084
132	GO:0071073	0.084
133	GO:0032412	0.084
134	GO:0060514	0.084
135	GO:0019102	0.084
136	GO:0045720	0.084
137	GO:0070974	0.084
138	GO:0060520	0.084
139	GO:0051940	0.084
140	GO:0051584	0.084
141	GO:0006940	0.084
142	GO:0007188	0.084
143	GO:0004941	0.084
144	GO:0007270	0.084
145	GO:0007212	0.083
146	GO:0002032	0.083
147	GO:0007195	0.083
148	GO:0022892	0.083
149	GO:0007208	0.083
150	GO:0071886	0.083
151	GO:0001994	0.083
152	GO:0061180	0.083

153	GO:0035239	0.083
154	GO:0060562	0.083
155	GO:0008289	0.083
156	GO:0042310	0.082
157	GO:0001591	0.082
158	GO:0004940	0.082
159	GO:0003061	0.082
160	GO:0031943	0.082
161	GO:0031279	0.082
162	GO:0051482	0.082
163	GO:0007190	0.082
164	GO:0055023	0.082
165	GO:0061051	0.082
166	GO:0033604	0.082
167	GO:0060134	0.082
168	GO:1900133	0.081
169	GO:0022898	0.081
170	GO:0015837	0.081
171	GO:0010513	0.081
172	GO:0000981	0.081
173	GO:0007626	0.081
174	GO:0015844	0.081

175	GO:0038052	0.081
176	GO:0008277	0.081
177	GO:1900135	0.081
178	GO:0051932	0.08
179	GO:0014827	0.08
180	GO:0030814	0.08
181	GO:0018993	0.08
182	GO:0033267	0.08
183	GO:0043266	0.08
184	GO:0045823	0.08
185	GO:0014831	0.08
186	GO:0004952	0.08
187	GO:0035112	0.079
188	GO:0048808	0.079
189	GO:0060685	0.079
190	GO:0005261	0.079
191	GO:0002001	0.079
192	GO:0071071	0.079
193	GO:0001547	0.079
194	GO:0034703	0.079
195	GO:0070472	0.079
196	GO:0010511	0.079

197	GO:0060442	0.079
198	GO:0030284	0.079
199	GO:0005215	0.079
200	GO:0032413	0.079
201	GO:0051953	0.079
202	GO:0043679	0.079
203	GO:0030817	0.079
204	GO:0060527	0.079
205	GO:0060526	0.079
206	GO:0045112	0.079
207	GO:0043568	0.079
208	GO:0060513	0.078
209	GO:0006171	0.078
210	GO:0004713	0.078
211	GO:0007628	0.078
212	GO:0035240	0.078
213	GO:0034776	0.078
214	GO:0030522	0.078
215	GO:0048386	0.078
216	GO:0004936	0.078
217	GO:0060750	0.078
218	GO:0060601	0.078

219	GO:0004882	0.078
220	GO:0007187	0.078
221	GO:0060571	0.078
222	GO:0045945	0.078
223	GO:0060525	0.078
224	GO:0043278	0.078
225	GO:0044306	0.078
226	GO:0045113	0.078
227	GO:0045726	0.078
228	GO:0015874	0.078
229	GO:0030594	0.078
230	GO:0007205	0.077
231	GO:0002209	0.077
232	GO:0001662	0.077
233	GO:0035640	0.077
234	GO:0034765	0.077
235	GO:0007189	0.077
236	GO:0040014	0.077
237	GO:0032410	0.077
238	GO:0001975	0.077
239	GO:0030534	0.077
240	GO:0034056	0.077

241	GO:0032414	0.077
242	GO:0070852	0.077
243	GO:0071502	0.076
244	GO:0070471	0.076
245	GO:0048243	0.076
246	GO:0000272	0.076
247	GO:0090030	0.076
248	GO:0060456	0.076
249	GO:0045777	0.076
250	GO:1901386	0.076
251	GO:0003084	0.076
252	GO:0009152	0.076
253	GO:0050805	0.076
254	GO:0034220	0.075
255	GO:0010459	0.075
256	GO:0014854	0.075
257	GO:0007285	0.075
258	GO:0004930	0.075
259	GO:0043949	0.075
260	GO:0002026	0.075
261	GO:0060523	0.075
262	GO:0060073	0.075

263	GO:0030054	0.075
264	GO:0008013	0.075
265	GO:0019199	0.075
266	GO:0001964	0.075
267	GO:0048242	0.075
268	GO:0052652	0.075
269	GO:0009190	0.075
270	GO:0033145	0.075
271	GO:0033148	0.075
272	GO:0032409	0.075
273	GO:0060737	0.075
274	GO:0010700	0.075
275	GO:0006359	0.075
276	GO:0046545	0.075
277	GO:0033602	0.074
278	GO:0055025	0.074
279	GO:0014061	0.074
280	GO:0004714	0.074
281	GO:0046676	0.074
282	GO:0015276	0.074
283	GO:0022834	0.074
284	GO:0032811	0.074

285	GO:0051588	0.074
286	GO:0001587	0.074
287	GO:0014060	0.074
288	GO:0051580	0.074
289	GO:0009260	0.074
290	GO:0014821	0.074
291	GO:0046873	0.074
292	GO:0003044	0.074
293	GO:0003025	0.074
294	GO:0051349	0.073
295	GO:0031281	0.073
296	GO:0051586	0.073
297	GO:0051944	0.073
298	GO:0051582	0.073
299	GO:0004888	0.073
300	GO:0002027	0.073
301	GO:0070473	0.073
302	GO:0006265	0.073
303	GO:0005887	0.073
304	GO:0031226	0.073
305	GO:0004937	0.072
306	GO:0004938	0.072

307	GO:0071883	0.072
308	GO:0001978	0.072
309	GO:0048384	0.072
310	GO:0032228	0.072
311	GO:0001541	0.072
312	GO:0071880	0.072
313	GO:0060160	0.072
314	GO:0060372	0.072
315	GO:2001257	0.072
316	GO:0090325	0.072
317	GO:0003918	0.072
318	GO:0061505	0.072
319	GO:0031692	0.071
320	GO:0030521	0.071
321	GO:0021894	0.071
322	GO:0021830	0.071
323	GO:0021853	0.071
324	GO:0007200	0.071
325	GO:0035625	0.071
326	GO:0014072	0.071
327	GO:0060421	0.071
328	GO:0002009	0.071

329	GO:0006164	0.071
330	GO:0060004	0.071
331	GO:0071882	0.071
332	GO:0071881	0.071
333	GO:0071878	0.071
334	GO:0060135	0.071
335	GO:0030315	0.071
336	GO:0003916	0.071
337	GO:0086003	0.071
338	GO:0086015	0.071
339	GO:0086018	0.071
340	GO:0008585	0.07
341	GO:0007638	0.07
342	GO:0086012	0.07
343	GO:0006937	0.07
344	GO:0031987	0.07
345	GO:0048754	0.07
346	GO:0035815	0.07
347	GO:0046777	0.07
348	GO:0048755	0.07
349	GO:0008022	0.07
350	GO:0004716	0.07

351	GO:0051208	0.069
352	GO:0010996	0.069
353	GO:0030432	0.069
354	GO:0035624	0.069
355	GO:0007267	0.069
356	GO:0051954	0.069
357	GO:0050681	0.069
358	GO:0051966	0.069
359	GO:0003012	0.069
360	GO:0086070	0.069
361	GO:0032795	0.069
362	GO:0060572	0.069
363	GO:0019838	0.069
364	GO:0046390	0.069
365	GO:0007171	0.069
366	GO:0048241	0.069
367	GO:0001963	0.069
368	GO:0086019	0.069
369	GO:0050955	0.069
370	GO:0008306	0.069
371	GO:0022890	0.068
372	GO:0060384	0.068

373	GO:0005244	0.068
374	GO:0022832	0.068
375	GO:0042698	0.068
376	GO:0004672	0.068
377	GO:0060068	0.068
378	GO:0033555	0.068
379	GO:0016773	0.068
380	GO:0016301	0.068
381	GO:0016772	0.068
382	GO:0030238	0.068
383	GO:0045741	0.068
384	GO:0001985	0.068
385	GO:1900273	0.068
386	GO:2001258	0.068
387	GO:0035264	0.068
388	GO:0008016	0.068
389	GO:0006812	0.068
390	GO:0007530	0.068
391	GO:0030819	0.068
392	GO:0016740	0.068
393	GO:0031623	0.067
394	GO:0001976	0.067

395	GO:0042383	0.067
396	GO:0050961	0.067
397	GO:0050965	0.067
398	GO:0014829	0.067
399	GO:0072522	0.067
400	GO:0050877	0.067
401	GO:0060159	0.067
402	GO:0005057	0.067
403	GO:0022843	0.067
404	GO:0086065	0.066
405	GO:0008324	0.066
406	GO:0014075	0.066
407	GO:0044260	0.066
408	GO:0019035	0.066
409	GO:0045870	0.066
410	GO:0009330	0.066
411	GO:0070474	0.066
412	GO:0050880	0.066
413	GO:0035150	0.066
414	GO:0042659	0.066
415	GO:0030431	0.066
416	GO:0045202	0.066

417	GO:0022602	0.066
418	GO:0032148	0.066
419	GO:0001659	0.066
420	GO:0090257	0.066
421	GO:0001504	0.066
422	GO:0035637	0.066
423	GO:0035249	0.066
424	GO:0002028	0.066
425	GO:0030540	0.065
426	GO:0043268	0.065
427	GO:0007613	0.065
428	GO:0046885	0.065
429	GO:0090066	0.065
430	GO:0033143	0.065
431	GO:0032553	0.065
432	GO:0032279	0.065
433	GO:0051823	0.065
434	GO:0007266	0.065
435	GO:0021769	0.065
436	GO:0003018	0.065
437	GO:0030554	0.065
438	GO:0060688	0.065

439	GO:0017076	0.065
440	GO:0032555	0.065
441	GO:0031224	0.065
442	GO:0033146	0.065
443	GO:0001820	0.064
444	GO:0035825	0.064
445	GO:0007131	0.064
446	GO:0003015	0.064
447	GO:0060047	0.064
448	GO:0030816	0.064
449	GO:0032549	0.064
450	GO:0001882	0.064
451	GO:0050998	0.064
452	GO:0032225	0.064
453	GO:0032550	0.064
454	GO:0001883	0.064
455	GO:0035639	0.064
456	GO:0018108	0.064
457	GO:0018212	0.064
458	GO:0032467	0.064
459	GO:0048729	0.064
460	GO:0008333	0.064

461	GO:0030539	0.064
462	GO:0048385	0.064
463	GO:0038023	0.064
464	GO:0043170	0.064
465	GO:0061458	0.064
466	GO:0016247	0.064
467	GO:0097195	0.063
468	GO:0014062	0.063
469	GO:0060751	0.063
470	GO:0051590	0.063
471	GO:1900271	0.063
472	GO:0035258	0.063
473	GO:0005524	0.063
474	GO:0030804	0.063
475	GO:0060406	0.063
476	GO:0021892	0.063
477	GO:0060065	0.063
478	GO:0044456	0.063
479	GO:0008344	0.063
480	GO:1901385	0.063
481	GO:0006312	0.063
482	GO:0001046	0.063

483	GO:0031696	0.063
484	GO:0030424	0.063
485	GO:0032559	0.063
486	GO:1902305	0.063
487	GO:0046058	0.063
488	GO:0030799	0.063
489	GO:0007600	0.062
490	GO:0034762	0.062
491	GO:0046488	0.062
492	GO:2000273	0.062
493	GO:0030810	0.062
494	GO:1900373	0.062
495	GO:0005021	0.062
496	GO:0042428	0.062
497	GO:1901019	0.062
498	GO:0000712	0.062
499	GO:0007608	0.062
500	GO:0051307	0.062
